# Whole-Genome Sequence of a Stenotrophomonas maltophilia Isolate from Tap Water in an Intensive Care Unit

**DOI:** 10.1128/mra.00995-22

**Published:** 2023-01-12

**Authors:** Ummu Afeera Zainulabid, Shing Wei Siew, Siti Munirah Musa, Sharmeen Nellisa Soffian, Petrick Periyasamy, Hajar Fauzan Ahmad

**Affiliations:** a Department of Internal Medicine, Kulliyyah of Medicine, International Islamic University Malaysia, Kuantan, Pahang, Malaysia; b Medical Department, Faculty of Medicine, Universiti Kebangsaan Malaysia, Cheras, Kuala Lumpur, Malaysia; c Faculty of Industrial Sciences and Technology, Universiti Malaysia Pahang, Gambang, Pahang, Malaysia; Loyola University Chicago

## Abstract

Here, we present a 4,508,936-bp complete genome sequence of Stenotrophomonas maltophilia strain HW002Y, which was isolated from the tap water in an intensive care unit at Sultan Ahmad Shah Medical Centre at the International Islamic University of Malaysia (Kuantan, Pahang, Malaysia). Sequencing was performed using a Nanopore Flongle flow cell.

## ANNOUNCEMENT

The Gram-negative bacillus Stenotrophomonas maltophilia is a multidrug-resistant organism that commonly infects patients in intensive care units. The source of contact is from environmental water sources, such as hospital tap water, direct contact, ingestion, aspiration, or aerosolization (1, 2). A sample was isolated from the hospital tap water in an intensive care unit at Sultan Ahmad Shah Medical Centre at the International Islamic University of Malaysia (IIUM) (Kuantan, Pahang, Malaysia). Three liters of tap water was vacuum filtered to concentrate the bacteria. The membrane filter was transferred to a collection tube containing phosphate-buffered saline, vortex-mixed, and left to soak overnight at room temperature. Subsequently, 0.1 mL of the solution was taken and cultured overnight at 37°C on ampicillin-containing Luria-Bertani agar. A presumed colony of S. maltophilia was selected, and the genomic DNA (gDNA) of the strain was extracted as described previously, using the GenElute bacterial gDNA kit (Sigma-Aldrich Co., St. Louis, MO, USA) (3). A high-sensitivity kit (DeNovix, Wilmington, DE) and agarose gel electrophoresis were used to evaluate the concentration and quality of the gDNA, respectively. Prior to library preparation, DNA was fragmented to an average length of >6 kb using SPRIselect reagent (Beckman Coulter Inc., Indianapolis, IN, USA) as described previously (4). Briefly, 1.25 μg of DNA was used for library preparation with a ligation sequencing kit (SQK-LSK110; Oxford Nanopore Technologies, Oxford, UK). The prepared library was then loaded onto a Flongle flow cell (R9.4.1; Oxford Nanopore Technologies) for 72 h. Base calling of raw data was performed using Guppy v6.0.6 (super accurate model). The raw read length of the genome is 12,000 reads and the *N*_50_ value is 14,704 bp. The base-called reads were length filtered to include only reads with a minimum read length of 3 kb, followed by *de novo* assembly using Flye v2.9 (nano-hq model) (5). The assembled genome was subsequently polished with one round with Racon v1.4.3 (https://github.com/isovic/racon) and then one round with medaka v1.6.0 (https://github.com/nanoporetech/medaka) (6), generating the final consensus assembly, which consists of one circular contig, as assessed by Flye v2.9 (7). HW002Y has a total length of 4,508,936 bp, with an average GC content of 66.6% and genome coverage of 231.0×. The percent identity between the query sequences and the bacterial species discovered with autoMLST (8) was examined via the Average Nucleotide Identity (ANI) Calculator v1.0 (https://www.ezbiocloud.net/tools/ani) (9), which showed that the sequence was 97.97% identical to the S. maltophilia reference genome (GenBank assembly accession number GCF_001274655). Due to the high level of nucleotide identity, it is therefore proven that the isolated bacterial sample is S. maltophilia. Genome annotation was carried out using the NCBI Prokaryotic Genome Annotation Pipeline (PGAP) v6.2 with the GeneMarkS2 v1.14_1.25 method (10), which resulted in one contig with 4,151 coding sequences and 85 RNAs. Unless otherwise noted, default parameters were used for all software tools.

The study was approved by the IIUM Kulliyyah of Medicine Research Committee (KRC) (research identification number 801) and the Department of Education and Research, Sultan Ahmad Shah Medical Centre, IIUM (registration number IISR22-06).

### Data availability.

The complete genome sequencing reads for Stenotrophomonas maltophilia HW002Y are available in the NCBI SRA under accession number SRR19579117 and BioProject accession number PRJNA847001. The GenBank accession number is CP104169.1, and the assembly accession number is GCA_025200825.1.
